# Host‐Specific Vocal Similarity in Fledgling Levaillant's Cuckoos and Babblers in West Africa

**DOI:** 10.1002/ece3.73330

**Published:** 2026-03-26

**Authors:** Tomás Redondo, Derek Engelbrecht, Isaac Kilusu, Clive R. Barlow

**Affiliations:** ^1^ Estación Biológica de Doñana CSIC Sevilla Spain; ^2^ Department of Biodiversity, Department of Biodiversity University of Limpopo Mankweng South Africa; ^3^ Village Weaver Birding Arusha Tanzania; ^4^ Birds of the Gambia, Brusubi Gardens Brufut The Gambia

**Keywords:** brood parasitism, *Clamator*, coevolution, cuckoo, *Turdoides*, vocal mimicry

## Abstract

Vocal mimicry in brood parasites may enhance fledgling survival by facilitating care or social acceptance by hosts. We quantified vocal similarity between fledgling Levaillant's cuckoos ( *Clamator levaillantii* ) and host fledglings using recordings from two host associations in West Africa and a small comparative set from South Africa. We distinguished two context‐defined classes of begging calls: food‐transfer calls produced during feeding events and non‐transfer calls produced outside food transfer. Multivariate acoustic measurements combined with discriminant and classification analyses showed that non‐transfer calls of cuckoos associated with the primary West African host, the Brown Babbler ( *Turdoides plebejus* ), closely matched non‐transfer calls of host fledglings. In contrast, food‐transfer calls were broadly similar across host species and showed little evidence of host‐specific vocal similarity in any association. Cuckoos associated with a secondary West African host, the Blackcap Babbler ( *T. reinwardtii* ), showed weak or no host‐specific vocal similarity overall, although one individual produced a distinctive non‐transfer call type that resembled host non‐transfer calls. South African material was sparse and revealed no clear host‐specific vocal similarity. Because the hosts compared are closely related babblers occupying similar habitats, the restriction of vocal similarity to a single association is unlikely to reflect general ecological convergence or shared constraints on vocal production. Instead, our findings are most consistent with host‐mediated selection acting after fledging, potentially favouring calls that promote tolerance or social acceptance within cooperatively breeding host groups. We also report new observations of host aggression towards fledgling cuckoos, highlighting the potential importance of vocal cues during the post‐fledging period.

## Graphical abstract

Young cuckoos can mimic host calls to gain care, but the extent of this similarity varies across hosts. Analysing fledgling begging calls across four host associations, we found that calls given during food transfer were broadly similar and did not match host calls, whereas cuckoos reared by their main West African host closely mimicked host begging non‐transfer calls. This pattern suggests that vocal mimicry may help fledgling cuckoos integrate into cooperative host groups.

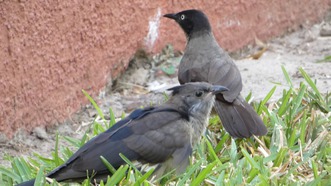

## 1 Introduction

Brood parasites such as cuckoos lay their eggs in the nests of other species, thereby exploiting host parental care and imposing fitness costs on their hosts (Soler 2017; Dillenseger 2024). This antagonistic interaction has driven a coevolutionary arms race, with hosts evolving defences—such as rejecting foreign eggs or nestlings—and parasites developing traits that increase their chances of successful exploitation (Davies 2011; Kilner and Langmore 2011). While most research has focused on adaptations at the egg stage (Davies 2011), far less is known about how parasite chicks promote acceptance and elicit appropriate care after hatching (Grim 2017; Rojas Ripari et al. 2021). Interactions at the chick stage therefore represent a major and still poorly understood gap in studies of host–parasite coevolution (Grim 2007). This bias is particularly pronounced during the fledgling period, despite increasing evidence that host–parasite conflicts and selection pressures often extend well beyond nest departure (Feeney, Welbergen, and Langmore 2014; De Mársico et al. 2018; Tyller et al. 2018).

In some host–parasite systems, parasitic chicks resemble host offspring in visual traits such as general appearance (Langmore et al. 2011; Attisano et al. 2023), mouth colouration and postural begging displays (Payne and Payne 2002; Jamie et al. 2020) or vocalisations (Jamie and Kilner 2018). In certain cases, this similarity reflects evolved mimicry driven by host rejection of dissimilar chicks (Noh et al. 2018, 2021), while in others, it may serve to enhance host exploitation by better matching (tuning into) host parental preferences (Langmore and Spottiswoode 2012). By tapping into established parent–offspring communication channels (Davies 2011), parasitic nestlings may elicit appropriate care from foster parents and avoid underfeeding or neglect. Functionally, mimicry and tuning may be equivalent from the parasite's perspective, and distinguishing between them can be conceptually difficult (Grim 2013; Dalziell et al. 2015). A recent review suggests that vocal similarity between parasite and host calls may be more widespread than previously appreciated. Jamie and Kilner (2018) found that more than half of parasitic species with described begging calls show some degree of vocal similarity to their hosts, with independent evolution of vocal similarity in at least six of seven parasitic avian clades (see also Rojas Ripari et al. 2019; McClean 2020). The forms of vocal similarity are also varied (Jamie and Kilner 2018): cuckoo chicks may resemble individual host nestlings (Tunheim et al. 2019; Noh et al. 2021), entire broods (Davies et al. 1998; Puswal et al. 2024) or even adult host calls (Jamie and de Silva 2014). However, rigorous quantitative evidence of vocal similarity remains scarce for many of these taxa (Jamie and Kilner 2018).

**FIGURE 1:**
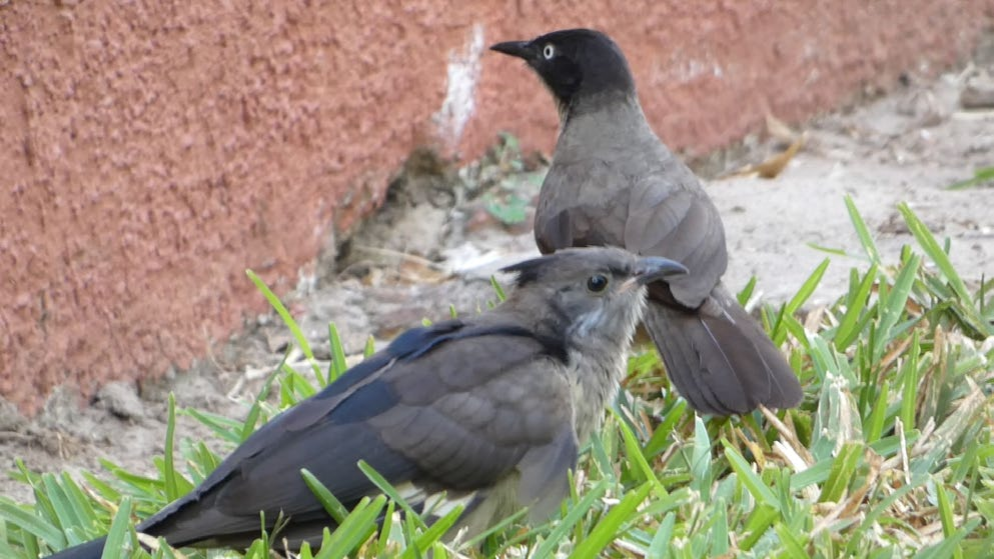
A fledgling Levaillant's cuckoo attended by Blackcap Babblers at Banjul, The Gambia (Photo credit: K. Hodgson).

One of the earliest and most frequently cited cases of putative vocal similarity between a parasitic chick and its host involves the Levaillant's Cuckoo ( *Clamator levaillantii* ), a species for which naturalists have long reported host‐like vocalisations by fledglings. Levaillant's Cuckoo is an intra‐African migrant that parasitises cooperatively breeding *Turdoides* babblers across much of sub‐Saharan Africa, although not all species in the genus are used (Irwin 1988; Erritzøe et al. 2012). Host use varies geographically: in south‐eastern Africa the primary host is the Arrow‐marked Babbler ( *Turdoides jardineii* ; 86% of records, *N* = 36), whereas in West Africa (Senegambia) the main host is the Brown Babbler ( *T. plebejus* ; 81%, *N* = 23), followed by the Blackcap Babbler ( *T. reinwardtii* ; 17%) (Barlow and Mann 2022) (Figure 1). In Zimbabwe, Levaillant's Cuckoo parasitised 7.8% of 217 Arrow‐marked Babbler nests (Payne and Payne 1967). Several features of this system point to a long history of host–parasite interaction. Levaillant's Cuckoo lays immaculate bluish eggs that closely resemble the eggs of most babbler hosts throughout its range (Erritzøe et al. 2012). Although egg rejection has not been documented (Steyn 1973), babblers frequently mob adult cuckoos and Arrow‐marked Babblers sometimes desert parasitised clutches (Steyn 1973). In Nigeria, Brown Babbler eggs show unusually high inter‐clutch colour variation that is finely matched by cuckoos, suggesting coevolution at the egg stage (Serle 1977; Stokke et al. 2002). After hatching, Levaillant's Cuckoo does not evict host eggs or chicks (Rowan 1983) but grows faster than its hosts (Steyn 1973; Jones 1992). Although newly hatched cuckoos and Arrow‐marked Babbler chicks have similar skin colouration, differences in gape morphology and colour increase with age, leading to growing visual mismatch (Steyn 1973; Rowan 1983). Despite this, fledglings are fed cooperatively by host groups for several weeks after leaving the nest (typically 3–5 weeks; Payne et al. 2021), a period during which vocal signals may be particularly important for maintaining host care. Against this background, early accounts from south‐eastern Africa described fledgling Levaillant's Cuckoos producing harsh, rattling or chattering calls strikingly similar to those of their babbler hosts (Vincent 1934; Jubb 1952; Benson and Pitman 1966). Later observations reported repetitive ‘ker, ker, ker’ contact calls resembling babbler food‐solicitation calls (Steyn 1973; Steyn and Howells 1975), although some authors suggested that cuckoos were imitating adult host calls rather than chick vocalisations (Vincent 1934; Jubb 1952; Mundy 1973; Vernon 1982). Notably, all such reports concern cuckoos parasitising Arrow‐marked Babblers or, in a single case, Hartlaub's Babbler ( *T. hartlaubii* ) (Benson and Pitman 1966).

Despite its historical prominence, the proposed case of vocal similarity in Levaillant's Cuckoo has important limitations. First, none of the published accounts are supported by quantitative sonographic analyses, making it difficult to evaluate the extent or structure of the reported vocal similarity (Jamie and Kilner 2018). Second, all descriptions concern a single host species, the Arrow‐marked Babbler, leaving open the possibility that similarity reflects shared habitat acoustics or other selective pressures unrelated to mimicry (Grim 2005; Dalziell et al. 2015). Robust evidence for mimicry requires demonstrating that parasite vocalisations vary across host associations and more closely resemble the calls of the host actually exploited than those of alternative hosts or conspecifics, as shown in *Vidua* finches and *Chalcites* cuckoos (Jamie et al. 2020; Attisano et al. 2025). Such a comparative framework is essential to assess whether vocal similarity reflects host‐driven coevolution, either to counter rejection or to exploit specific parental responses (Anderson et al. 2009; Attisano et al. 2025). Levaillant's Cuckoo offers a valuable opportunity to apply this approach because, in West Africa—its other major breeding region—it parasitises more than one babbler species. Based on long‐standing descriptions from south‐eastern Africa reporting vocal similarity between cuckoo fledglings and their babbler hosts, we predicted that West African cuckoos would more closely resemble their local host species than hosts from southern Africa. More specifically, if fledgling vocal similarity reflects an adaptive response to host behaviour, cuckoos raised by the main West African host should more closely match that host's calls than those of secondary hosts or cuckoos associated with other hosts. Accordingly, the objectives of this study were to (i) provide the first quantitative description of fledgling begging calls in Levaillant's Cuckoo across different host associations, and (ii) test whether cuckoo vocalisations are more similar to those of their actual hosts than to alternative hosts or other cuckoos.

## 2 Methods

### 2.1 Sound Recordings

Audio and video recordings of Levaillant's Cuckoo and host fledglings reared by Blackcap and Brown Babblers in The Gambia were collected or curated by C. R. Barlow between 2006 and 2025. Vocalisations of one cuckoo and two host fledglings associated with Arrow‐marked Babblers were recorded by D. Engelbrecht and I. Kilusu. We also included one recording of a Levaillant's cuckoo fledgling fed by a Kurrichane Thrush ( *Turdus libonyana* ) in South Africa (Tony Archer, XC418749, available at www.xeno‐canto.org/418749) (Archer 2012). No recordings were available for Kurrichane Thrush fledgling calls, but we included fledgling begging calls of the African Thrush ( *Turdus pelios* ), a closely related species (Batista et al. 2020), recorded by CRB in The Gambia. Because Kurrichane Thrushes are not established hosts of Levaillant's Cuckoo, this observation may reflect auxiliary feeding rather than rearing by this species (Sealy and Lorenzana 1997), and we therefore treat this record cautiously. The original audio files were annotated by the recordists to identify call types and behavioural context. A descriptive list of all sound files, collection dates and sites, and recording equipment is provided in Table S1.

### 2.2 Sound Analysis

All audio tracks were converted to WAV format using either VLC media player (VideoLAN 2024) or Adobe Premiere Pro (sample rate: 48,000 Hz; bitrate: 312 kb/s). Vocalisations were analysed in Avisoft‐SASLab Pro (Specht 2002). Annotated continuous spectrograms were generated for each recording to identify callers, describe behavioural context and classify vocal elements. Each element was defined by its distinct amplitude envelope; in cases where two successive elements formed a single vocalisation, they were analysed separately.

Fledgling calls were classified as either Food‐transfer calls, produced during food delivery by adults (Video S1), or Non‐transfer calls, given during the long intervals between feedings, accompanied by varying degrees of begging postures such as wing‐quivering or crouching (Videos S1 and S2). We deliberately avoid the terms host‐present and host‐absent begging (e.g., Grim 2008; Honza et al. 2018) because they are poorly defined in cooperatively breeding babblers. Because fledgling cuckoos accompany social groups of cooperative hosts, host presence is often continuous, making host‐present vs. host‐absent begging ambiguous; we therefore classified calls by feeding context rather than host proximity. Cuckoos reared by Blackcap Babblers produced two distinct types of non‐transfer calls, which were analysed separately: wide‐band, shorter Brief non‐transfer calls, similar in duration and bandwidth to Brief non‐transfer calls of other cuckoos and longer, narrower‐band Meow non‐transfer calls (one individual).

Recordings varied substantially in signal‐to‐noise ratio and type of low‐frequency background noise, although most calls exceeded 700 Hz. This was confirmed by analysing amplitude spectra of background segments adjacent to focal calls. To reduce noise interference, a 0.6 kHz high‐pass filter was applied to spectrograms. Spectrograms were generated using a 512‐point FFT with a FlatTop window, 100% frame size, 50% overlap, and a resulting bandwidth of 162 Hz. This provided a temporal resolution of 11.6 ms and frequency resolution of 43 Hz. For each selected element, non‐overlapping extraneous sounds were manually excluded, and amplitude thresholds were adjusted to enhance the signal‐to‐noise ratio. Heavily masked calls or recordings (e.g., SEN_1222_#1–#3) overlapping with other bird and insect sounds were excluded from analysis.

For each element, nine acoustic parameters were automatically extracted. These included: call duration and the mean and standard deviation of frequency bandwidth, entropy (a measure of energy dispersion across frequencies; pure tones have low entropy, broadband calls have high), and the first (Q25) and third (Q75) quartiles of energy distribution, which serve as robust estimators of low and high frequency boundaries, respectively (Fristrup and Watkins 1993). Most vocalisations consisted of harsh or multiband calls with low‐frequency modulation; therefore, spectral measurements were based on the mean spectrum of the entire element, following Specht (2002). We aimed to measure as many elements as possible. In total, 2552 vocal elements were analysed, although sample sizes varied considerably across species and call types (see Tables S2 and S3).

### 2.3 Statistical Analysis

All statistical analyses were conducted in R Statistical Software (R Core Team 2025). Following the approach of Jamie et al. (2020) and Attisano et al. (2025), vocal similarity was assessed using linear discriminant analysis (LDA) and multinomial logistic regression (MLR), implemented via the lda() function in the *MASS* package (v7.3‐65) and the multinom() function in the *nnet* package (v7.3‐20), respectively (Venables and Ripley 2002). While LDA assumes multivariate normality, it performs reliably with small sample sizes; MLR, by contrast, makes fewer distributional assumptions and serves as a non‐parametric alternative (Jamie et al. 2020).

Rather than averaging calls per individual, each measured call was treated as an independent data point, following Jamie et al. (2020). The nine acoustic variables were used as predictors to determine whether fledgling cuckoo calls more closely resembled those of their own host species, or instead those of other host species or cuckoos reared by different hosts. Two training datasets were created: one comprising calls from host species, and another including calls from the focal cuckoo's associated host and from non‐focal cuckoos associated with other hosts. These datasets were used to train the LDA and MLR models, which were then applied to classify the calls of focal cuckoo fledglings. Similarity between call types was assessed using confusion matrices derived from the LDA and MLR outputs (Jamie et al. 2020; Attisano et al. 2025).

To further evaluate the overall similarity across all call types between each cuckoo–host pair and alternative hosts or cuckoos, we calculated two binary performance metrics—F1 score and Matthews Correlation Coefficient (MCC)—by pooling all call types for each focal cuckoo–host pair. The F1 score is the harmonic mean of precision (proportion of predicted positives that are true positives) and recall (proportion of actual positives correctly classified), ranging from 0 (complete classification failure) to 1 (perfect classification). The MCC incorporates all four components of the confusion matrix (true/false positives and negatives), and it is considered particularly reliable for imbalanced datasets (Chicco and Jurman 2020). MCC values range from −1 (poor) to 1 (perfect), with 0 indicating random performance. Values of MCC > 0.3 are generally considered moderate and > 0.5 strong.

Differences in acoustic variables of Food‐transfer and non‐transfer begging calls between each cuckoo and its associated host, and among cuckoos associated with different hosts, were analysed using generalised linear mixed models (GLMMs), assuming a Gamma error distribution with a log link, using the *glmmTMB* package (v1.1.12) (Brooks et al. 2017). Species was included as a fixed effect and individual bird identity as a random intercept to account for repeated measures. Analyses were restricted to West African cuckoos and hosts because South African cuckoos were represented by a single individual. Inference focused on three a priori contrasts using the *emmeans* package (Lenth 2025): Blackcap Babbler hosts vs. cuckoos, Brown Babbler hosts vs. cuckoos and Blackcap Babbler cuckoos vs. Brown Babbler cuckoos. For each contrast we obtained estimated marginal means on the link scale and reported back‐transformed effects on the response scale as *ratios* (multiplicative differences) with 95% confidence intervals from which *Z* statistics were computed. *p*‐values were adjusted across the three planned contrasts within each variable using the Holm procedure.

### 2.4 Ethical Note

This study was purely observational and did not require ethical approval. Sound and video recordings were conducted with minimal disturbance to the birds, ensuring that their natural behaviour was not disrupted.

## 3 Results

### 3.1 Host Vocalisations

**FIGURE 2:**
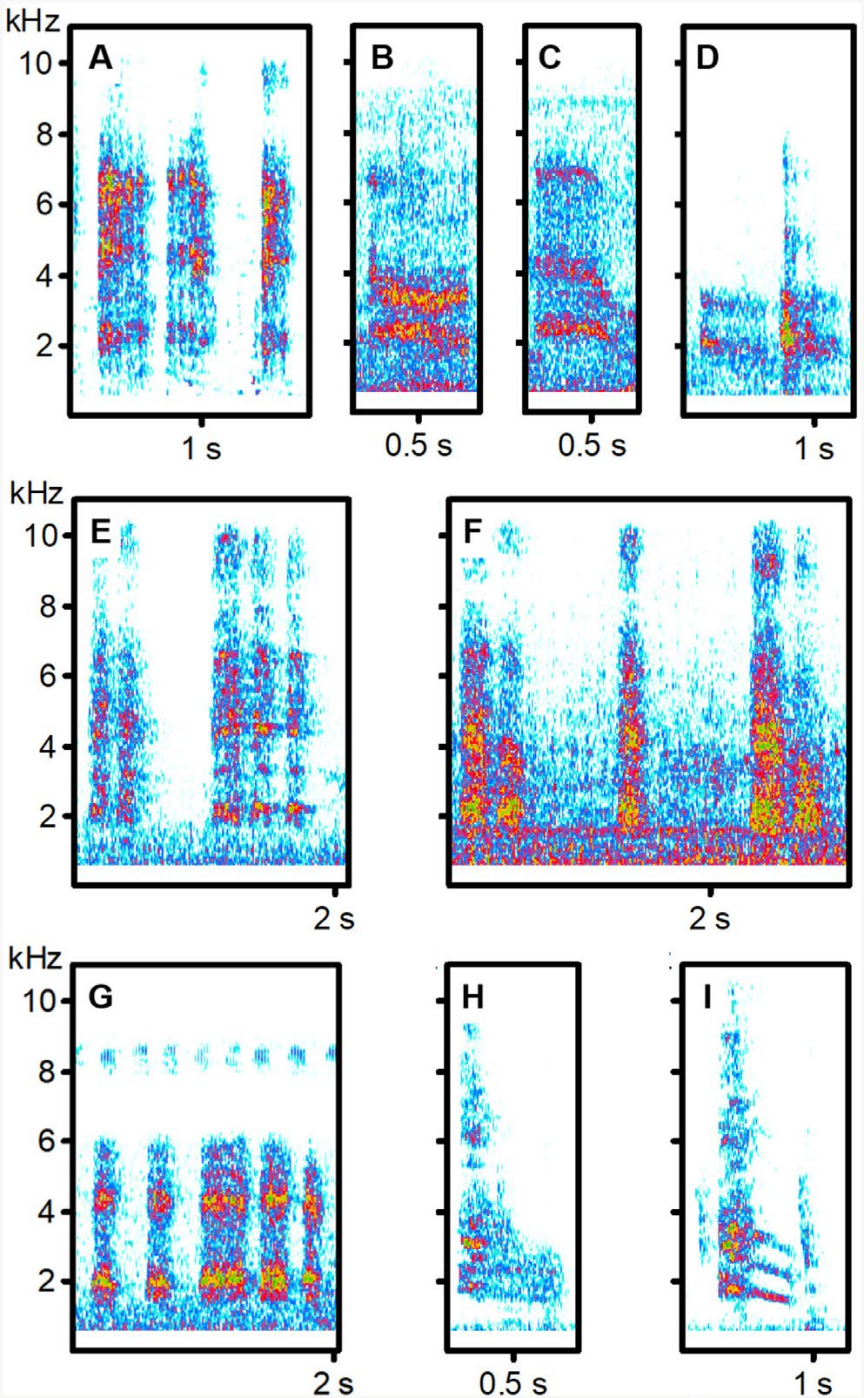
Begging calls of Blackcap Babbler hosts and cuckoos. (A) Host Food‐transfer calls (sound recording BRU_0322); (B, C) Non‐transfer calls from two hosts (BRU_0423, BRU_0524); (D) Two host Non‐transfer calls with a Food‐transfer call in between (BRU_0423); (E) Cuckoo Food‐transfer calls; (F) Brief non‐transfer calls including double calls by the same cuckoo (SEN_1222); (G) Brief non‐transfer calls by a different bird (KEN_1213); (H, I) Meow non‐transfer calls (SUK_1123). For details of sound recordings, see Table S1.

**FIGURE 3:**
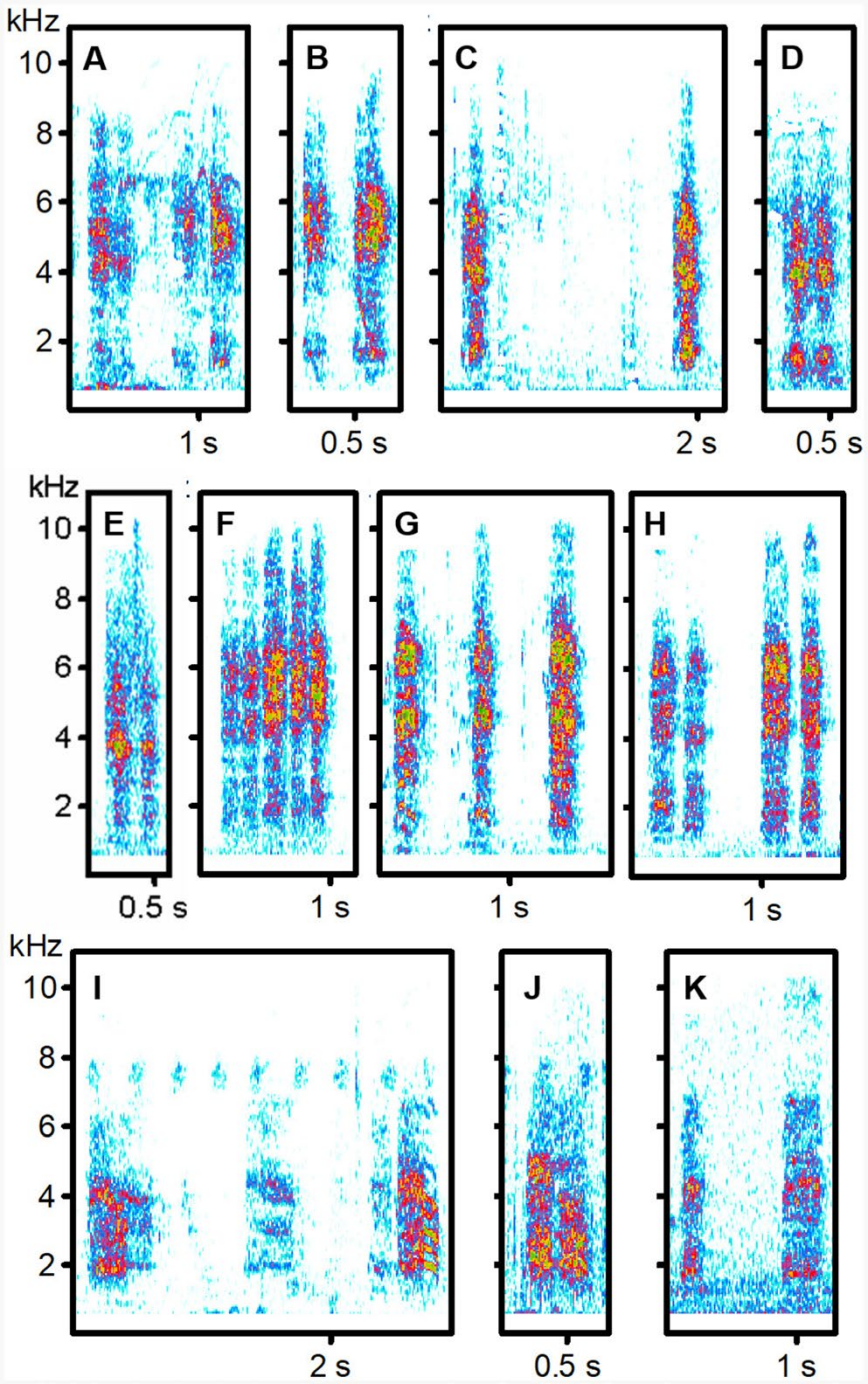
Begging calls of Brown Babbler host and cuckoo fledglings. (A, B) Food‐transfer calls from two hosts (sound recordings BRU_1021, BRU_0622); (C–E) Non‐transfer begging calls from two different hosts, including double calls (D, E) (BRU_0316, BRU_0722, BRU_0316); Cuckoo Food‐transfer (F) and Brief non‐transfer calls (G, H), including double calls from the same individual (BRU_0114); (I, J) Brief non‐transfer calls from a second cuckoo, including double calls (ABU_1206); (K) Brief non‐transfer calls from a third bird (SAB_0125). For details of sound recordings, see Table S1.

**FIGURE 4:**
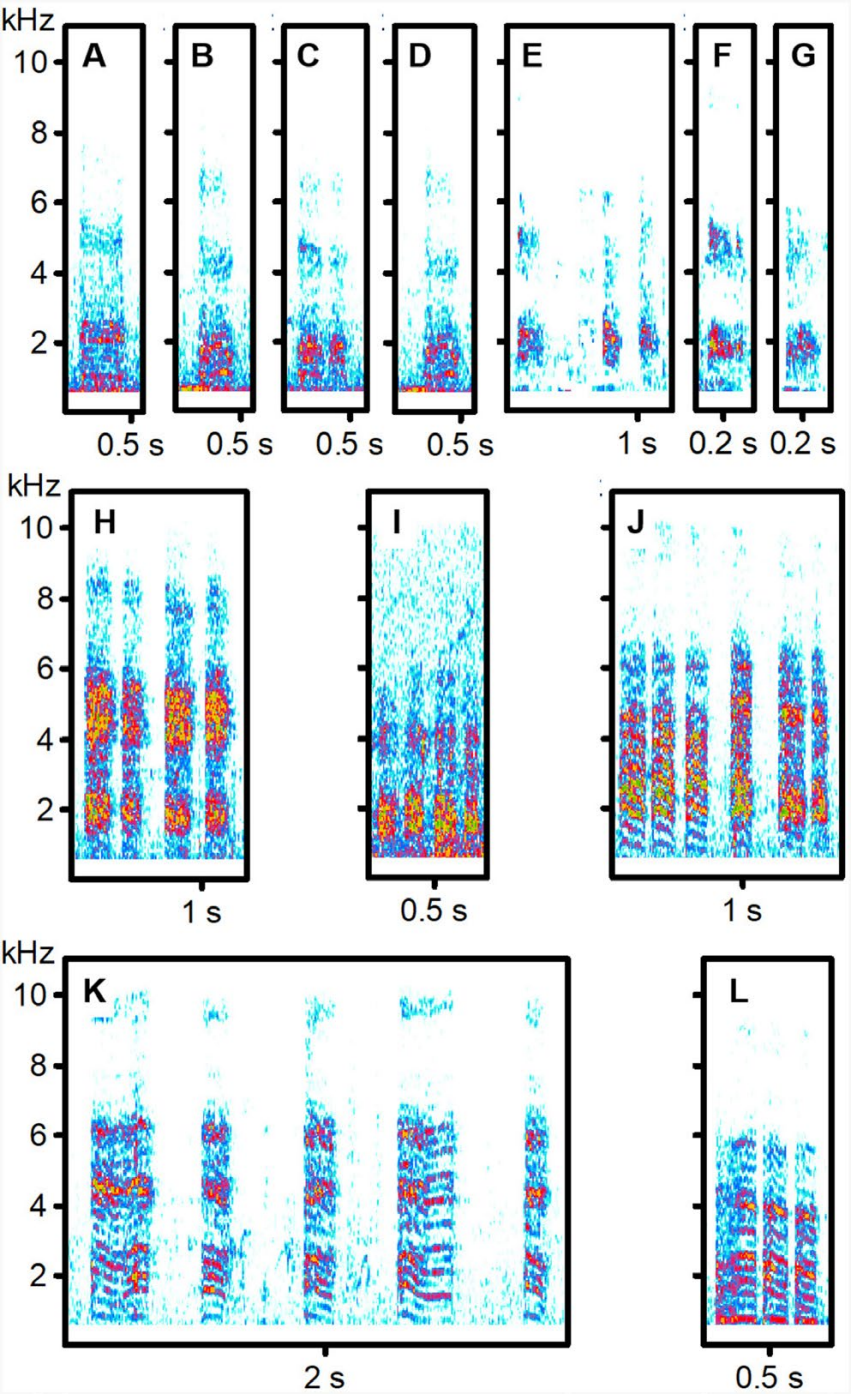
Begging calls of Arrow‐Marked Babbler hosts and cuckoo. (A–G) Non‐transfer begging calls of two hosts (A–D: Sound recording POL_0722; E–G: OLO_0723); (H, I) Cuckoo Food‐transfer calls; (J–L) Cuckoo Brief non‐transfer calls from the same bird (HOE_0424). For details of sound recordings, see Table S1.

**FIGURE 5:**
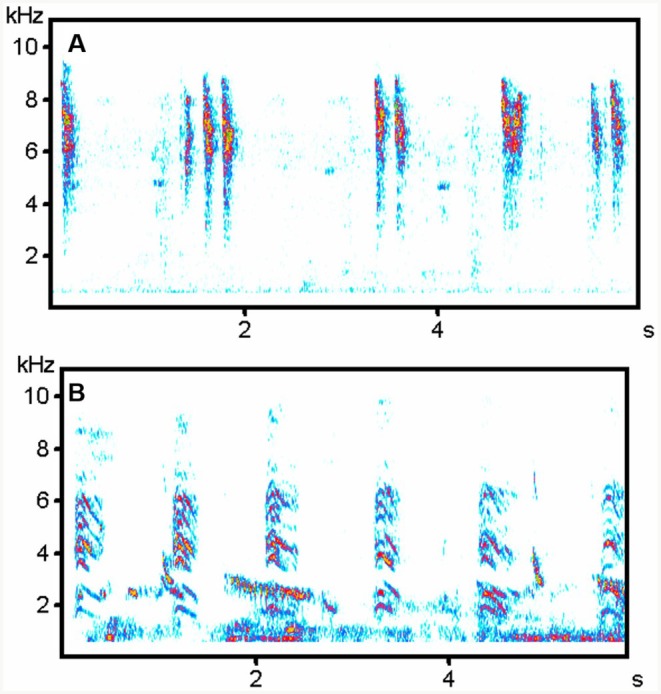
Begging non‐transfer calls of an African Thrush (A) (sound recording BRU_0822), and a Levaillant's cuckoo begging from a Kurrichane Thrush (B) (from a recording by Tony Archer (XC418749, www.xeno‐canto.org/418749), reproduced under a CC BY‐NC licence). For details of sound recordings, see Table S1.

We analysed fledgling calls from three babbler hosts and African Thrush, distinguishing Food‐transfer (recorded only for the West African babblers) and Non‐transfer calls (sample sizes in Table S2; examples in Figures 2, 3, 4, 5). Linear discriminant analysis (LDA) of host calls, classified by species and call type, correctly assigned 85.1% of vocal elements. African Thrush and Blackcap Babbler non‐transfer calls were the most distinctive (92% and 100% correctly classified, respectively), whereas food‐transfer calls of the two West African babblers showed the greatest overlap (misclassification: 28.6% for Blackcap Babblers and 12.4% for Brown Babblers; Table S2).

### 3.2 Cuckoo Vocalisations

Cuckoos vocalised almost continuously while accompanying their host groups (Videos S1 and S2). Interactions included host feeding (Video S1), cuckoos pecking at hosts (Video S3) and occasional host aggression towards cuckoos (Videos S3 and S4); however, hosts most often appeared to ignore cuckoo begging attempts (Video S2). We analysed non‐transfer begging calls from all cuckoos and food‐transfer begging calls from all babbler‐associated cuckoos (sample sizes in Table S3; examples in Figures 2, 3, 4, 5). LDA of cuckoo calls, classified by host and call type, correctly assigned 78.4% of vocal elements to their host‐associated group. Meow non‐transfer calls in the Blackcap Babbler‐associated cuckoo and Brief non‐transfer calls in Brown Babbler‐associated cuckoos were the most distinctive (91.2% and 90.8% correctly classified, respectively). By contrast, Food‐transfer and Brief non‐transfer calls of Blackcap Babbler‐associated cuckoos were frequently misclassified as Brown Babbler‐associated cuckoo calls (80.0% and 65.3% misclassified, respectively). For the Arrow‐marked Babbler‐associated cuckoo, 65.4% of Brief non‐transfer calls were correctly classified, but 80.0% of Food‐transfer calls were misclassified as Blackcap Babbler‐associated Brief non‐transfer calls. Only 15.0% of Brief non‐transfer calls from the Kurrichane Thrush‐associated cuckoo were correctly identified, with 45.0% misclassified as Arrow‐marked Babbler Brief non‐transfer calls (Table S3).

### 3.3 Similarity Between Calls of Parasites and Hosts

Only the Brief non‐transfer begging calls of Brown Babbler cuckoos were classified as more similar (90.9%) to their host than to calls of other hosts (Table 1). In contrast, their Food‐transfer calls were more similar (71%) to Food‐transfer calls of Blackcap Babbler hosts. When both call types were considered together, Brown Babbler cuckoos showed a high F1 score (0.70, average of LDA and MLR) but a low MCC coefficient (−0.06) (Table 1; Table S4). When both call types were compared specifically to the Non‐transfer begging calls of their host (i.e., excluding food‐transfer calls), Brown Babbler cuckoos showed a high F1 score (0.70, average of LDA and MLR) and a moderate MCC coefficient (0.31, average of LDA and MLR) (Table 2; Table S5), indicating greater similarity to their phylogenetically unrelated host than to other cuckoos. Among Blackcap Babbler cuckoos, only Meow non‐transfer calls showed moderate similarity (46.4%) to host Non‐transfer calls (Table 1), but this similarity was lower than that to calls of other cuckoos (Table 2). The remaining calls of Blackcap Babbler cuckoos, as well as those of cuckoos associated with other host species, were not particularly similar to their respective hosts (Table 1) and more closely resembled calls of cuckoos associated with other hosts (Table 2). All host‐associated groups except Brown Babbler‐associated cuckoos showed poor classification performance (F1 and MCC) with respect to their hosts, both in comparisons with other hosts (Table 1) and with other cuckoos (Table 2). Results from MLR analyses were consistent with those from LDA (Tables S4 and S5).

**TABLE 1:** Predicted similarity of begging calls based on the linear discriminant functions for each cuckoo–host pair. Entries show, for each call type and host association (rows), the number of cuckoo calls classified into each host call‐type category (columns). The F1 score and Matthews correlation coefficient (MCC) provide call‐type–independent measures of overall similarity between each cuckoo and its associated host relative to other hosts.

	Hosts						F1^a^	MCC^b^
	Arrow‐marked Babbler	Blackcap Babbler		Brown Babbler		African Thrush		
	Non‐transfer	Non‐transfer	Food‐transfer	Non‐transfer	Food‐transfer	Non‐transfer		
Arrow‐marked Babbler‐cuckoo								
Brief non‐transfer	9	18	0	389	17	0	0.04	0.01
Food‐transfer	0	0	0	8	1	0		
Blackcap Babbler‐cuckoos								
Brief non‐transfer	7	0	2	193	4	0	0.15	0.07
Food‐transfer	0	0	0	12	0	0		
Meow non‐transfer	23	32	0	14	0	0		
Brown Babbler‐cuckoos								
Brief non‐transfer	10	4	85	1293	29	2	0.78	0.06
Food‐transfer	1	0	22	8	0	0		
Kurrichane Thrush‐cuckoo								
Brief non‐transfer	0	0	0	5	2	0	0.00	0.00

^a^ 0 (complete classification failure) < F1 < 1 (perfect classification).

^b^ –1 (poor) < MCC < 1 (perfect), with 0 indicating random performance. MCC > 0.3 is often considered moderate and > 0.5 strong.

**TABLE 2:** Predicted similarity of begging calls based on the linear discriminant functions for each cuckoo–host pair. Entries show, for each call type and host association (rows), the number of cuckoo calls classified as their associated host or as cuckoos associated with other hosts (columns). The F1 score and Matthews correlation coefficient (MCC) provide call‐type–independent measures of similarity between each cuckoo and its associated host relative to other cuckoos.

	Associated host		Cuckoos								F1^a^	MCC^b^
			Arrow‐marked Babbler		Blackcap Babbler			Brown Babbler		Kurrichane Thrush		
	Brief non‐transfer	Food‐transfer	Brief non‐transfer	Food‐transfer	Brief non‐transfer	Food‐transfer	Meow non‐transfer	Brief non‐transfer	Food‐transfer	Brief non‐transfer		
Arrow‐marked Babbler‐cuckoo												
Brief non‐transfer	10	—	—	—	232	0	2	177	0	12	0.03	−0.16
Food‐transfer	1	—	—	—	7	0	0	1	0	0		
Blackcap Babbler‐cuckoos												
Brief non‐transfer	0	0	102	6	—	—	—	96	0	2	0.04	−0.15
Food‐transfer	1	0	1	0	—	—	—	10	0	0		
Meow non‐transfer	15	0	7	1	—	—	—	7	0	39		
Brown Babbler‐cuckoos												
Brief non‐transfer	986	43	165	0	216	0	3	—	—	10	0.75	0.32
Food‐transfer	28	2	1	0	0	0	0	—	—	0		
Kurrichane Thrush‐cuckoo												
Brief non‐transfer	0	—	2	0	0	0	1	4	0	—	0.00	−0.01

^a^ 0 (complete classification failure) < F1 < 1 (perfect classification).

^b^ –1 (poor) < MCC < 1 (perfect), with 0 indicating random performance. MCC > 0.3 is often considered moderate and > 0.5 strong.

**FIGURE 6:**
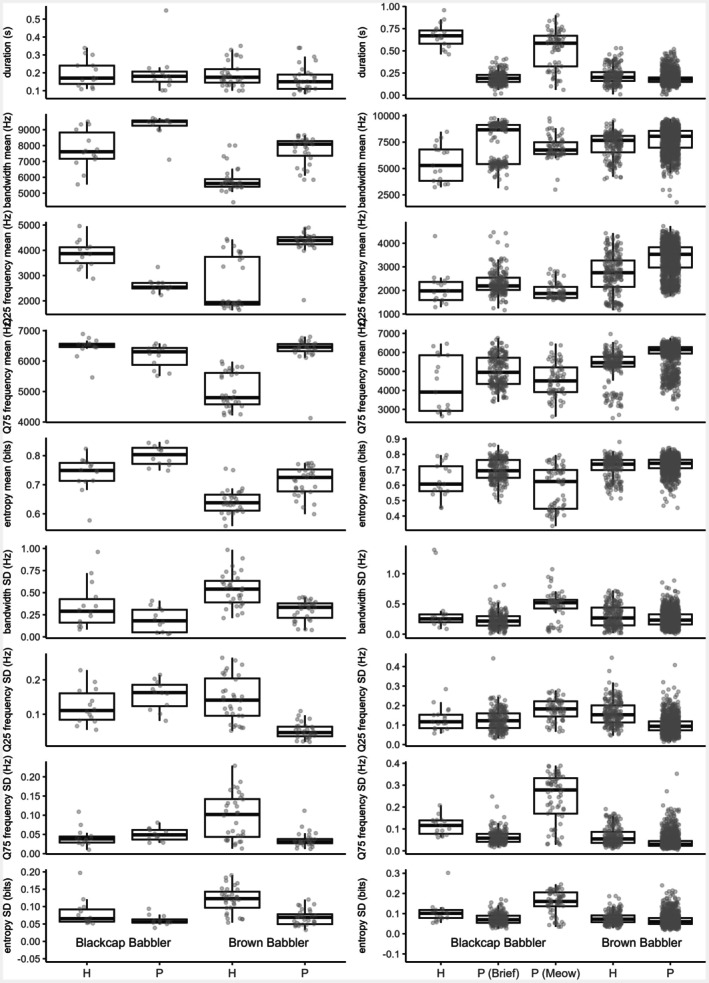
Distribution of acoustic variables—mean and standard deviation of frequency bandwidth across call time, call duration, entropy and lower (Q25) and higher (Q75) frequencies—for begging calls of West African hosts (H) and parasites (P), grouped by calling context. Left panel: Food‐transfer calls; right panel: Non‐transfer calls. Boxes show medians, interquartile ranges (IQR, hinges) and values within ±1.5 IQR (whiskers).

Comparison of acoustic features of West African calls supported these results. In Food‐transfer calls, differences between cuckoos and hosts were mainly explained by entropy and by within‐call variation in bandwidth and lower (Q25) frequencies (Figure 6; Table 3). For Brief non‐transfer calls, only lower‐frequency (Q25) differences separated Brown Babbler cuckoos from their hosts. In contrast, Brief non‐transfer calls of Blackcap Babbler cuckoos—indistinguishable from Brown Babbler cuckoo non‐transfer calls according to GLMM—differed from Blackcap Babbler host calls in both duration and temporal variation in bandwidth (Figure 5; Table 3). Meow calls of the Blackcap Babbler cuckoo differed from host non‐transfer calls only in Q25 frequency but contrasted with Brown Babbler cuckoo Brief non‐transfer calls in several features, including duration, temporal variation in bandwidth and entropy and both lower (Q25) and higher (Q75) frequencies (Figure 5; Table 3). Differences in Q25 frequencies should be taken cautiously, as they may reflect recording conditions, given the substantial variation in background low‐frequency noise across samples.

**TABLE 3:** Results of the generalised linear mixed models for the planned comparisons of begging call features between cuckoos and hosts from West Africa.

Call type/acoustic variable	Blackcap Babbler hosts vs. cuckoos			Brown Babbler hosts vs. cuckoos			Blackcap Babbler cuckoos vs. Brown Babbler cuckoos		
	Ratio	*z*	*p*	Ratio	*z*	*p*	Ratio	*z*	*p*
Food‐transfer calls									
Bandwidth (mean)	1.190	1.384	0.279	1.281	2.553	0.032	1.201	1.478	0.279
Bandwidth (SD)	0.551	−2.938	**0.007**	0.565	−4.382	**0.000**	0.639	−2.549	**0.011**
Duration	1.012	0.072	0.943	0.856	−1.222	0.635	1.210	1.249	0.635
Entropy (mean)	1.084	3.093	**0.002**	1.109	6.211	**0.000**	1.124	5.175	**0.000**
Entropy (SD)	0.744	−1.988	0.094	0.593	−3.124	**0.005**	0.887	−0.886	0.376
Lower (Q25) frequency (mean)	0.679	−1.111	0.533	1.284	0.926	0.533	0.603	−1.454	0.438
Lower (Q25) frequency (SD)	1.269	0.607	0.544	0.429	−2.715	**0.013**	3.033	2.882	**0.012**
Higher (Q75) frequency (mean)	0.953	−0.495	1.000	1.179	2.173	0.089	0.965	−0.370	1.000
Higher (Q75) frequency (SD)	1.170	0.266	1.000	0.681	−0.841	1.000	1.410	0.591	1.000
Brief non‐transfer calls									
Bandwidth (mean)	1.263	1.294	0.587	1.025	0.162	1.000	0.940	−0.333	1.000
Bandwidth (SD)	0.608	−2.456	**0.042**	0.876	−0.992	0.515	0.832	−1.132	0.515
Duration	0.288	−8.577	**0.000**	1.002	0.021	0.984	0.833	−1.405	0.320
Entropy (mean)	1.093	1.102	0.811	1.011	0.157	1.000	0.966	−0.408	1.000
Entropy (SD)	0.701	−2.364	0.054	0.970	−0.288	1.000	0.987	−0.099	1.000
Lower (Q25) frequency (mean)	1.055	0.225	1.000	0.980	−0.100	1.000	0.870	−0.563	1.000
Lower (Q25) frequency (SD)	0.934	−0.519	0.603	0.635	−5.751	**0.000**	1.207	1.988	0.094
Higher (Q75) frequency (mean)	1.153	0.756	1.000	1.001	0.005	1.000	0.935	−0.342	1.000
Higher (Q75) frequency (SD)	0.543	−1.829	0.202	1.055	0.198	1.000	0.965	−0.108	1.000
Meow non‐transfer calls									
Bandwidth (mean)	1.287	1.079	0.562				0.957	−0.183	0.855
Bandwidth (SD)	1.303	1.155	0.248				1.783	2.937	**0.007**
Duration	0.769	−1.397	0.162				2.228	4.490	**0.000**
Entropy (mean)	0.924	−0.761	0.447				0.816	−1.918	0.110
Entropy (SD)	1.454	1.774	0.076				2.056	3.667	**0.000**
Lower (Q25) frequency (mean)	0.920	−0.305	0.761				0.758	−0.989	0.645
Lower (Q25) frequency (SD)	1.341	1.918	0.055				1.733	4.418	**0.000**
Higher (Q75) frequency (mean)	1.078	0.280	1.000				0.873	−0.492	1.000
Higher (Q75) frequency (SD)	2.136	1.883	0.060				3.804	3.335	**0.002**

*Note:* Values in bold indicate significant differences (*p* < 0.05).

## 4 Discussion

This study presents the first quantitative assessment of vocal similarity between fledgling Levaillant's Cuckoos and their hosts, revealing differences among host‐associated groups. Cuckoos associated with their primary West African host, the Brown Babbler, produced begging calls—especially non‐transfer calls—that closely resembled those of host fledglings, relative to other hosts and cuckoos. In contrast, food‐transfer calls were broadly similar across host species, and cuckoos raised by secondary hosts, including Blackcap Babblers, showed little or no host‐specific similarity. Evidence from South African cuckoos was limited and revealed no clear acoustic matching with hosts.

Vocalisations associated with fledgling care were complex, with different call types used depending on species and context (e.g., food‐transfer or non‐transfer), as reported in other babblers (Yambem et al. 2021) and cuckoos (Payne and Payne 1998; Grim 2008; Honza et al. 2018). Our classification analyses effectively distinguished between host and cuckoo calls based on type and species using time and spectral variables commonly applied in similar studies and based on comparable sample sizes (Anderson et al. 2009; Ranjard et al. 2010; De Mársico et al. 2012; Jamie et al. 2020; Attisano et al. 2025). While we used methods designed to tolerate unbalanced datasets, our sample was uneven, particularly in the number of vocal elements per host–cuckoo pair. Brown Babbler calls were best represented in number, though the number of individuals was comparable across West African hosts. Nevertheless, classification performance did not appear to be strongly biassed by sample size. For instance, Meow calls of a Blackcap Babbler cuckoo were correctly matched to host Non‐transfer calls—those most similar in sonograms—despite limited data (Table 1). Similarly, the greater abundance of Arrow‐marked Babbler host and cuckoo calls, relative to Blackcap Babblers, did not result in misclassification of other cuckoo calls (Table 2; Tables S2 and S3).

Among West African babblers, fledgling food‐transfer calls were similar across species and resembled Brown Babbler non‐transfer calls, but not those of Blackcap Babblers. Only cuckoos reared by Brown Babblers produced calls that consistently resembled vocalisations of their host—especially non‐transfer calls. In contrast, Blackcap Babbler cuckoos showed no such match, though one individual gave distinctive Meow non‐transfer calls that resembled host calls (Tables 1 and 3) but were more similar to those of other host‐associated cuckoos (Table 2). Actually, Brief non‐transfer calls of Blackcap Babbler cuckoos were very similar to calls of Brown Babbler cuckoos. South African data were fragmentary, lacking recordings from Kurrichane Thrushes and food‐transfer calls from Arrow‐marked Babblers. Available evidence showed no clear acoustic matching between South African cuckoos and their putative hosts. The Kurrichane Thrush record may actually reflect auxiliary feeding by a non‐foster bird (Barlow and Mann 2022; Sealy and Lorenzana 1997). Many calls given by the two South African cuckoos had a harmonic structure (Figures 3 and 4), resembling the cat‐like *nyaaa* described in East Africa (Payne et al. 2021). These differed from the Brief, harsh non‐transfer calls of West African cuckoos (Figures 1 and 2) but sounded, to the human ear, similar to the Meow calls of Blackcap Babbler cuckoos (Figure 1, Table 2).

Vocal similarity between Brown Babbler cuckoos and their fledgling hosts is unlikely to arise from general ecological convergence or from by‐products of vocal learning—two common explanations for interspecific similarity (Grim 2005; Dalziell et al. 2015)—because Brown and Blackcap Babblers share similar breeding habits and habitats (Collar and Robson 2020a, 2020b), yet vocal similarity was detected only in cuckoos associated with the former. Although this system meets several conceptual and methodological criteria for mimicry—such as clear identification of model and signal and greater similarity to the focal host than to alternative hosts (Jamie et al. 2020)—direct evidence of a selective mechanism imposed by the receiver is lacking and will require targeted experimental and developmental studies (Jamie et al. 2020; de Jager and Anderson 2019). Moreover, the restriction of vocal similarity to a single cuckoo–host association, together with the absence of documented host discrimination against parasitic nestlings or fledglings, warrants a cautious interpretation. Rather than supporting a fully established mimicry system, our results are consistent with the possibility that vocal similarity in Brown Babbler cuckoos reflects either (i) selection for a more generalised call that is effective across several babbler species with broadly similar begging repertoires (Sealy and Lorenzana 1997), or (ii) host‐mediated selection acting at the fledgling stage, potentially favouring calls that promote tolerance or social acceptance within cooperative host groups (De Mársico et al. 2012; Grim 2006, 2017). In the following sections, we consider ecological and behavioural factors that may help explain why such similarity is expressed in one host association but not in others.

Vocal similarity of Brown Babblers by Levaillant's Cuckoos may reflect a coevolutionary arms race, as suggested by the presence of presumably coadapted traits such as clutch desertion, potential egg mimicry and variation in host egg appearance (Kilner and Langmore 2011; see Section 1). Theory predicts that strong egg defences can inhibit the evolution of later‐stage adaptations (the rarer‐enemy effect; Grim 2006). Consequently, chick discrimination is expected mainly in hosts that accept at least some parasite eggs (supported by available data; Grim 2017), unless host–parasite interactions are both frequent and long‐standing (Britton et al. 2007)—conditions likely met in this system. In The Gambia, cuckoos occurred in 19% of Brown Babbler groups (*N* = 37) and 17% of Blackcap Babbler groups with fledglings (*N* = 17) (CR Barlow, unpubl. data, 2005–2025), potentially exceeding parasitism rates reported for Zimbabwe (Payne and Payne 1967). Both cuckoos and Brown Babblers favour open savanna woodlands (Collar and Robson 2020b; Payne et al. 2021), a climatically stable habitat that may have promoted long‐term coevolution (Spottiswoode et al. 2012). Levaillant's Cuckoos do not evict host chicks, but host chicks rarely survive to fledging—only 1 in 20 (5%) parasitised groups recorded in The Gambia contained both host and cuckoo fledglings (CR Barlow, unpubl. data; after Barlow and Mann 2022), which indicates a high virulence (Rasmussen and Sealy 2006; Ridley and Thompson 2012). Also, year‐round cooperative breeding by Brown Babblers may buffer the costs of recognition errors, facilitating the evolution of chick discrimination (Langmore et al. 2003). A broadly comparable context is provided by the Australian Horsfield's Bronze‐cuckoo (*Chalcites basalis*), one of the best‐documented cases of host‐specific begging‐call mimicry in brood parasites (Payne and Payne 1998; Langmore et al. 2008). This generalist evictor parasitises multiple host species and exhibits host‐dependent plasticity in chick vocalisations, with nestlings matching calls of their primary host and adjusting them when reared by secondary hosts (Langmore et al. 2008; Feeney, Stoddard, et al. 2014). Although laying strategies in Levaillant's cuckoo remain unknown, comparable patterns of multi‐host parasitism occur in other *Clamator* species. Females of the closely related Great Spotted Cuckoo ( *Clamator glandarius* ), for example, parasitise more than one corvid host simultaneously (Baglione et al. 2016). Their chicks produce host‐like begging calls at the fledgling stage, but not earlier in development (Mundy 1973; Redondo and de Arias Reyna 1988; Roldán et al. 2013). As in Levaillant's cuckoos, fledglings are reared by cooperative host groups and remain associated with them for several weeks after fledging (Soler et al. 1995; Baglione and Canestrari 2017), a prolonged interaction thought to favour selection for recognition and mimicry (Grim 2006, 2017; De Mársico et al. 2012). However, unlike hosts of the Horsfield's Bronze‐cuckoo, there is currently no evidence that Levaillant's cuckoo hosts discriminate on the basis of vocal cues (Langmore et al. 2003; Colombelli‐Négrel et al. 2012).

Levaillant's cuckoos reared by their primary West African host, the Brown Babbler, showed clear vocal similarity to host calls, whereas cuckoos raised by a secondary host did not. As in the bronze‐cuckoo system, the absence of mimicry in some host associations may reflect either constraints on call plasticity or weak or absent selection by hosts. In Horsfield's Bronze‐cuckoos, for instance, fledglings reared by a secondary host, the Scarlet Robin ( *Petroica multicolor* ), fail to match the robin's broad‐band calls and instead produce calls resembling those given when reared by other hosts (Payne and Payne 1998). Similarly, Blackcap Babbler cuckoos in our study produced non‐transfer calls more similar to those of Brown Babbler cuckoos than to those of their foster host, although one individual produced Meow calls closely matching Blackcap Babbler non‐transfer calls, indicating that imitation is at least possible. An alternative explanation is that neither Scarlet Robins nor Blackcap Babblers discriminate (or disfavour) against non‐mimetic calls and thus exert little pressure to reinforce or select for vocal similarity. While some hosts preferentially respond to species‐specific calls when provisioning or allocating food (Villain et al. 2015; Yasukawa et al. 2016; Ursino et al. 2018), others respond indiscriminately (Vidas‐Guscic et al. 2024), or even favour traits absent from their own young (Gloag and Kacelnik 2013). Vocal similarity between parasites and hosts appears widespread (Jamie and Kilner 2018), yet direct evidence of host rejection based on vocal cues remains rare (De Mársico et al. 2018).

Alternatively, vocal similarity may offer benefits beyond food acquisition by fledglings. Cooperative babblers use complex vocal systems to coordinate foraging and predator defence (Raihani and Ridley 2007; Radford and Ridley 2008; Yambem et al. 2021), with different affiliative calls sharing similar acoustic features (Crane et al. 2016; Yambem et al. 2021). In Jungle Babblers (*Argya striata*), fledglings emit “chack” calls that are acoustically similar to adult contact calls that maintain group cohesion (Yambem et al. 2021). Cuckoo Brief non‐transfer calls could serve a comparable affiliative role, promoting social integration and facilitating access to food, protection and learning opportunities within the babbler group (Feeney et al. 2013; Baglione and Canestrari 2017). This role may be particularly important during the fledgling stage, when parasites begin to resemble adult cuckoos and hosts may become reluctant to feed—or may even attack—them (Somanader 1946; Sanjeeva Raj 1964; Gaston 1976; Vernon 1982; Soler et al. 2014; Redondo 1993; Fawcett and Fawcett 2020). To previous reports of Arrow‐marked Babblers attacking fledgling cuckoos they were provisioning (Steyn and Howells 1975; Barry 1998; Irwin 1988), we add two new observations from The Gambia (Table S1; Videos S3 and S4). Communal attacks by large hosts such as babblers or corvids may be particularly effective in expelling cuckoos from the host group. In birds, recognition of conspecifics often involves integrating multisensory cues and categorising vocalisations through species‐specific predispositions (Matyjasiak 2005; Louder et al. 2019). Frequent production of calls perceived as affiliative may help cuckoos reduce aggression and maintain host tolerance—potentially explaining why cuckoo fledglings reared by cooperative and/or large‐sized hosts produce vocalisations resembling adult host calls (Jamie and de Silva 2014).

## Author Contributions

**Tomás Redondo:** conceptualization (equal), data curation (equal), formal analysis (lead), funding acquisition (equal), methodology (lead), project administration (supporting), resources (equal), software (lead), supervision (supporting), validation (equal), visualization (lead), writing – original draft (lead), writing – review and editing (equal). **Derek Engelbrecht:** data curation (equal), funding acquisition (equal), investigation (equal), resources (equal), validation (equal), writing – review and editing (equal). **Isaac Kilusu:** data curation (equal), funding acquisition (equal), investigation (equal), validation (equal), writing – review and editing (equal). **Clive R. Barlow:** conceptualization (equal), data curation (lead), funding acquisition (equal), investigation (lead), methodology (lead), project administration (lead), resources (equal), supervision (lead), validation (equal), writing – original draft (supporting), writing – review and editing (equal).

## Funding

This work was supported by Ministerio de Ciencia, Innovación y Universidades (PID2021‐126673NB‐100). Additional funds were provided by Severo Ochoa Program GrantCEX2024‐001498‐S funded by MICIU/AEI/10.13039/501100011033.

## Conflicts of Interest

The authors declare no conflicts of interest.

## Supporting information


**Table S1:** Complete list of sound recordings of Levaillant's cuckoo and babbler host fledglings.
**Table S2:** Predicted similarity among host begging calls based on linear discriminant analysis.
**Table S3:** Predicted similarity among cuckoo begging calls based on linear discriminant analysis.
**Table S4:** Predicted similarity of begging calls based on the logistic regression functions for each cuckoo–host pair.
**Table S5:** Predicted similarity of begging calls based on the logistic regression functions for each cuckoo–host pair.

**Video S1:** Food‐transfer and non‐transfer begging calls produced by a Levaillant's cuckoo fledgling associated with Blackcap Babblers (Credits: K. Hodgson).

**Video S2:** Food‐transfer and non‐transfer begging calls produced by a Levaillant's cuckoo fledgling associated with Brown Babblers (Credits: A. Brohaugh).

**Video S3:** Aggression by Brown Babblers towards a fledgling Levaillant's cuckoo (Credits: A. Brohaugh).

**Video S4:** Aggression by Blackcap Babblers towards a fledgling Levaillant's cuckoo (Credits: M. Oskampf).

## Data Availability

Analyses reported in this article can be reproduced using the original audio and video recordings, data and R code available at Mendeley Data: Redondo et al. (2026). https://data.mendeley.com/datasets/b4fmtw95yy/4.
