# Supplementary material for: Host‐Specific Vocal Similarity in Fledgling Levaillant's Cuckoos and Babblers in West Africa
Source: Ecol Evol. 2026 Mar 26;16(4):e73330. doi: 10.1002/ece3.73330 (PMC13107273; doi:10.1002/ece3.73330)
Supplement: Supplementary file 1 — Table S1: Complete list of sound recordings of Levaillant's cuckoo and babbler host fledglings. Table S2: Predicted similarity among host begging calls based on linear discriminant analysis. Table S3: Predicted similarity among cuckoo begging calls based on linear discriminant analysis. Table S4: Predicted similarity of begging calls based on the logistic regression functions for each cuckoo–host pair. Table S5: Predicted similarity of begging calls based on the logistic regression functions for each cuckoo–host pair. [file ECE3-16-e73330-s002.docx]

**SUPPLEMENTARY MATERIAL**

**Host-specific vocal similarity in fledgling Levaillant’s Cuckoos and babblers in West Africa**

Tomás Redondo^a^, Derek Engelbrecht^b^, Isaac Kilusu^c^ & Clive R. Barlow^d^†

*a Estación Biológica de Doñana (CSIC), Sevilla, Spain*

*b Department of Biodiversity, University of Limpopo, Sovenga, 0727, South Africa*

*c Arusha, Tanzania*

*d Birds of The Gambia, Brusubi Gardens, Brufut, The Gambia*

*† Deceased. Contributions to this article were made before his death*

**Table S1.** Complete list of sound recordings of Levaillant’s cuckoo and babbler host fledglings. Good-quality recordings included in the acoustic analyses are highlighted in red; * indicates recordings annotated by the recordist. Different entries for the same individual/site/date correspond to separate sound files. ML = Macaulay Library, Cornell University, Ithaca, USA (macaulaylibrary.org); XC = Xeno-canto (xenocanto.org). All original recordings are available at <https://data.mendeley.com/datasets/b4fmtw95yy/4>

| **ID** | **Species** | **Host race** | **Site of recording** | **Date (d/m/y)** | **Recordist** | **Equipment** | **File type** | **Description** |
| --- | --- | --- | --- | --- | --- | --- | --- | --- |
| HOE_0424_1 | *Clamator levaillantii* | *Turdoides jardineii* | Hoedspruit Wildlife Estate, Limpopo (South Africa) | 01/04/2024 | Derek Engelbrecht | Canon EOS R6 camera | audio WAV | One fledgling + 6–7 hosts (Macaulay Library (ML617040041, ML617040040, ML617040028) |
| HOE_0424_2 | *Clamator levaillantii* | *Turdoides jardineii* | Hoedspruit Wildlife Estate, Limpopo (South Africa) | 01/04/2024 | Derek Engelbrecht | Canon EOS R6 camera | audio WAV | One fledgling + 6–7 hosts (Macaulay Library (ML617040041, ML617040040, ML617040028) |
| HOE_0424_3 | *Clamator levaillantii* | *Turdoides jardineii* | Hoedspruit Wildlife Estate, Limpopo (South Africa) | 01/04/2024 | Derek Engelbrecht | Canon EOS R6 camera | audio WAV | One fledgling + 6–7 hosts (Macaulay Library (ML617040041, ML617040040, ML617040028) |
| HOE_0424_4 | *Clamator levaillantii* | *Turdoides jardineii* | Hoedspruit Wildlife Estate, Limpopo (South Africa) | 01/04/2024 | Derek Engelbrecht | Canon EOS R6 camera | audio WAV | One fledgling + 6–7 hosts (Macaulay Library (ML617040041, ML617040040, ML617040028) |
| HOE_0424_5 | *Clamator levaillantii* | *Turdoides jardineii* | Hoedspruit Wildlife Estate, Limpopo (South Africa) | 01/04/2024 | Derek Engelbrecht | Canon EOS R6 camera | audio WAV | One fledgling + 6–7 hosts (Macaulay Library (ML617040041, ML617040040, ML617040028) |
| HOE_0424_6 | *Clamator levaillantii* | *Turdoides jardineii* | Hoedspruit Wildlife Estate, Limpopo (South Africa) | 01/04/2024 | Derek Engelbrecht | Canon EOS R6 camera | audio WAV | One fledgling + 6–7 hosts (Macaulay Library (ML617040041, ML617040040, ML617040028) |
| HOE_0424_7 | *Clamator levaillantii* | *Turdoides jardineii* | Hoedspruit Wildlife Estate, Limpopo (South Africa) | 01/04/2024 | Derek Engelbrecht | Canon EOS R6 camera | video MP4 | One fledgling + 6–7 hosts (Macaulay Library (ML617040041, ML617040040, ML617040028) |
| HOE_0424_8 | *Clamator levaillantii* | *Turdoides jardineii* | Hoedspruit Wildlife Estate, Limpopo (South Africa) | 01/04/2024 | Derek Engelbrecht | Canon EOS R6 camera | audio WAV | One fledgling + 6–7 hosts (Macaulay Library (ML617040041, ML617040040, ML617040028) |
| ABU_1206_1 | *Clamator levaillantii* | *Turdoides plebejus* | Abuko Nature Reserve (Gambia) | 09/12/2006 | Clive R. Barlow* | Sony TCM cassette recorder | audio MP3 | Two fledglings (well-grown tails) + 4 hosts (Barlow & Mann 2022) |
| ABU_1206_2 | *Clamator levaillantii* | *Turdoides plebejus* | Abuko Nature Reserve (Gambia) | 09/12/2006 | Clive R. Barlow* | Sony TCM cassette recorder | audio MP3 | Two fledglings (well-grown tails) + 4 hosts (Barlow & Mann 2022) |
| ABU_1206_3 | *Clamator levaillantii* | *Turdoides plebejus* | Abuko Nature Reserve (Gambia) | 09/12/2006 | Clive R. Barlow* | Sony TCM cassette recorder | audio MP3 | Two fledglings (well-grown tails) + 4 hosts (Barlow & Mann 2022) |
| ABU_1206_4 | *Clamator levaillantii* | *Turdoides plebejus* | Abuko Nature Reserve (Gambia) | 09/12/2006 | Clive R. Barlow* | Sony TCM cassette recorder | audio MP3 | Two fledglings (well-grown tails) + 4 hosts (Barlow & Mann 2022) |
| ABU_1206_5 | *Clamator levaillantii* | *Turdoides plebejus* | Abuko Nature Reserve (Gambia) | 09/12/2006 | Clive R. Barlow* | Sony TCM cassette recorder | audio MP3 | Two fledglings (well-grown tails) + 4 hosts (Barlow & Mann 2022) |
| ABU_1206_6 | *Clamator levaillantii* | *Turdoides plebejus* | Abuko Nature Reserve (Gambia) | 09/12/2006 | Clive R. Barlow* | Sony TCM cassette recorder | audio MP3 | Two fledglings (well-grown tails) + 4 hosts (Barlow & Mann 2022) |
| ABU_1206_7 | *Clamator levaillantii* | *Turdoides plebejus* | Abuko Nature Reserve (Gambia) | 09/12/2006 | Clive R. Barlow* | Sony TCM cassette recorder | audio MP3 | Two fledglings (well-grown tails) + 4 hosts (Barlow & Mann 2022) |
| ABU_1206_8 | *Clamator levaillantii* | *Turdoides plebejus* | Abuko Nature Reserve (Gambia) | 09/12/2006 | Clive R. Barlow* | Sony TCM cassette recorder | audio MP3 | Two fledglings (well-grown tails) + 4 hosts (Barlow & Mann 2022) |
| ABU_1206_9 | *Clamator levaillantii* | *Turdoides plebejus* | Abuko Nature Reserve (Gambia) | 09/12/2006 | Clive R. Barlow* | Sony TCM cassette recorder | audio MP3 | Two fledglings (well-grown tails) + 4 hosts (Barlow & Mann 2022) |
| ABU_1206_10 | *Clamator levaillantii* | *Turdoides plebejus* | Abuko Nature Reserve (Gambia) | 09/12/2006 | Clive R. Barlow* | Sony TCM cassette recorder | audio MP3 | Two fledglings (well-grown tails) + 4 hosts (Barlow & Mann 2022) |
| ABU_1206_11 | *Clamator levaillantii* | *Turdoides plebejus* | Abuko Nature Reserve (Gambia) | 09/12/2006 | Clive R. Barlow | Sony hand camera | video MPG | Two fledglings (well-grown tails) + 4 hosts (Barlow & Mann 2022) |
| BRU_0114_1 | *Clamator levaillantii* | *Turdoides plebejus* | Brusubi (Gambia) | 04/01/2014 | Clive R. Barlow* | Marantz PMD 660, SennheiserK6 microphone, Rode 21/22 windshield | audio WAV | One fledgling + 5 hosts (Barlow & Mann 2022) |
| BRU_0114_2 | *Clamator levaillantii* | *Turdoides plebejus* | Brusubi (Gambia) | 04/01/2014 | Clive R. Barlow* | Marantz PMD 660, SennheiserK6 microphone, Rode 21/22 windshield | audio WAV | One fledgling + 5 hosts (Barlow & Mann 2022) |
| BRU_0114_3 | *Clamator levaillantii* | *Turdoides plebejus* | Brusubi (Gambia) | 04/01/2014 | Clive R. Barlow* | Marantz PMD 660, SennheiserK6 microphone, Rode 21/22 windshield | audio WAV | One fledgling + 5 hosts (Barlow & Mann 2022) |
| BRU_0114_4 | *Clamator levaillantii* | *Turdoides plebejus* | Brusubi (Gambia) | 04/01/2014 | Clive R. Barlow* | Marantz PMD 660, SennheiserK6 microphone, Rode 21/22 windshield | audio WAV | One fledgling + 5 hosts (Barlow & Mann 2022) |
| BRU_0114_5 | *Clamator levaillantii* | *Turdoides plebejus* | Brusubi (Gambia) | 06/01/2014 | Clive R. Barlow* | Marantz PMD 660, SennheiserK6 microphone, Rode 21/22 windshield | audio WAV | One fledgling + 5 hosts (Barlow & Mann 2022) |
| BRU_0114_6 | *Clamator levaillantii* | *Turdoides plebejus* | Brusubi (Gambia) | 06/01/2014 | Clive R. Barlow* | Marantz PMD 660, SennheiserK6 microphone, Rode 21/22 windshield | audio WAV | One fledgling + 5 hosts (Barlow & Mann 2022) |
| BRU_0114_7 | *Clamator levaillantii* | *Turdoides plebejus* | Brusubi (Gambia) | 08/01/2014 | Clive R. Barlow* | Marantz PMD 660, SennheiserK6 microphone, Rode 21/22 windshield | audio WAV | One fledgling + 5 hosts (Barlow & Mann 2022) |
| BRU_0114_8 | *Clamator levaillantii* | *Turdoides plebejus* | Brusubi (Gambia) | 08/01/2014 | Clive R. Barlow* | Marantz PMD 660, SennheiserK6 microphone, Rode 21/22 windshield | audio WAV | One fledgling + 5 hosts (Barlow & Mann 2022) |
| BRU_0114_9 | *Clamator levaillantii* | *Turdoides plebejus* | Brusubi (Gambia) | 12/01/2014 | Clive R. Barlow* | Marantz PMD 660, SennheiserK6 microphone, Rode 21/22 windshield | audio WAV | One fledgling + 5 hosts (Barlow & Mann 2022) |
| BRU_0114_10 | *Clamator levaillantii* | *Turdoides plebejus* | Brusubi (Gambia) | 13/01/2014 | Clive R. Barlow* | Marantz PMD 660, SennheiserK6 microphone, Rode 21/22 windshield | audio WAV | One fledgling + 5 hosts (Barlow & Mann 2022) |
| SAB_0125_1 | *Clamator levaillantii* | *Turdoides plebejus* | Sabi (Eastern Gambia) | 01/01/2025 | Asaph Brohaugh | Olympus OM-D E-M1 camera, microphone Shenggu SG108 | video MP4 | One fledgling + 5 hosts (sometimes showing aggression) |
| SAB_0125_2 | *Clamator levaillantii* | *Turdoides plebejus* | Sabi (Eastern Gambia) | 01/01/2025 | Asaph Brohaugh | Olympus OM-D E-M1 camera, microphone Shenggu SG108 | video MP4 | One fledgling + 5 hosts (sometimes showing aggression) |
| SAB_0125_3 | *Clamator levaillantii* | *Turdoides plebejus* | Sabi (Eastern Gambia) | 01/01/2025 | Asaph Brohaugh | Olympus OM-D E-M1 camera, microphone Shenggu SG108 | video MP4 | One fledgling + 5 hosts (sometimes showing aggression) |
| SAB_0125_4 | *Clamator levaillantii* | *Turdoides plebejus* | Sabi (Eastern Gambia) | 01/01/2025 | Asaph Brohaugh | Olympus OM-D E-M1 camera, microphone Shenggu SG108 | video MP4 | One fledgling + 5 hosts (sometimes showing aggression) |
| SAB_0125_5 | *Clamator levaillantii* | *Turdoides plebejus* | Sabi (Eastern Gambia) | 03/01/2025 | Asaph Brohaugh | Olympus OM-D E-M1 camera, microphone Shenggu SG108 | video MP4 | One fledgling + 5 hosts (sometimes showing aggression) |
| SAB_0125_6 | *Clamator levaillantii* | *Turdoides plebejus* | Sabi (Eastern Gambia) | 03/01/2025 | Asaph Brohaugh | Olympus OM-D E-M1 camera, microphone Shenggu SG108 | video MP4 | One fledgling + 5 hosts (sometimes showing aggression) |
| SAB_0125_7 | *Clamator levaillantii* | *Turdoides plebejus* | Sabi (Eastern Gambia) | 03/01/2025 | Asaph Brohaugh | Olympus OM-D E-M1 camera, microphone Shenggu SG108 | video MP4 | One fledgling + 5 hosts (sometimes showing aggression) |
| SAB_0125_8 | *Clamator levaillantii* | *Turdoides plebejus* | Sabi (Eastern Gambia) | 03/01/2025 | Asaph Brohaugh | Olympus OM-D E-M1 camera, microphone Shenggu SG108 | video MP4 | One fledgling + 5 hosts (sometimes showing aggression) |
| KEN_1213_1 | *Clamator levaillantii* | *Turdoides reinwardtii* | Keneba (Gambia) | 03/12/2013 | Tony Fulford* | Zoom H4n recorder with Rode NTG2 shotgun microphone | audio MP3 | One fledgling + 4 hosts (Barlow & Mann 2022) |
| KEN_1213_2 | *Clamator levaillantii* | *Turdoides reinwardtii* | Keneba (Gambia) | 07/12/2013 | Tony Fulford* | Zoom H4n recorder with Rode NTG2 shotgun microphone | audio MP4 | One fledgling + 4 hosts (Barlow & Mann 2022) |
| SEN_1222_1 | *Clamator levaillantii* | *Turdoides reinwardtii* | Senegambia Beach (Gambia) | 02/12/2022 | Karen & Robin Hodgson | Lumix DC-FZ82 hand camera | video MP4 | One fledgling + several hosts Masked by background insect sound |
| SEN_1222_2 | *Clamator levaillantii* | *Turdoides reinwardtii* | Senegambia Beach (Gambia) | 02/12/2022 | Karen & Robin Hodgson | Lumix DC-FZ82 hand camera | video MP4 | One fledgling + several hosts. Masked by background insect sound |
| SEN_1222_3 | *Clamator levaillantii* | *Turdoides reinwardtii* | Senegambia Beach (Gambia) | 02/12/2022 | Karen & Robin Hodgson | Lumix DC-FZ82 hand camera | video MP4 | One fledgling + several hosts. Masked by background insect sound |
| SEN_1222_4 | *Clamator levaillantii* | *Turdoides reinwardtii* | Senegambia Beach (Gambia) | 06/12/2022 | Karen & Robin Hodgson | Lumix DC-FZ82 hand camera | video MP4 | One fledgling + several hosts |
| SEN_1222_5 | *Clamator levaillantii* | *Turdoides reinwardtii* | Senegambia Beach (Gambia) | 06/12/2022 | Karen & Robin Hodgson | Lumix DC-FZ82 hand camera | video MP4 | One fledgling + several hosts |
| SEN_1222_6 | *Clamator levaillantii* | *Turdoides reinwardtii* | Senegambia Beach (Gambia) | 06/12/2022 | Karen & Robin Hodgson | Lumix DC-FZ82 hand camera | video MP4 | One fledgling + several hosts |
| SUK_1123_1 | *Clamator levaillantii* | *Turdoides reinwardtii* | Sukuta (Gambia) | 30/10/2023 | Myrthe Oskampf* | Galaxy A51 phone | audio M4A | One fledgling (near full-tail) + 2 hosts |
| SUK_1123_2 | *Clamator levaillantii* | *Turdoides reinwardtii* | Sukuta (Gambia) | 30/10/2023 | Myrthe Oskampf* | Galaxy A51 phone | audio M4A | One fledgling (near full-tail) + 2 hosts |
| SUK_1123_4 | *Clamator levaillantii* | *Turdoides reinwardtii* | Sukuta (Gambia) | 30/10/2023 | Myrthe Oskampf | Galaxy A51 phone | video MP4 | One fledgling (near full-tail) + 2 hosts |
| SUK_1123_5 | *Clamator levaillantii* | *Turdoides reinwardtii* | Sukuta (Gambia) | 30/10/2023 | Myrthe Oskampf* | Galaxy A51 phone | audio M4A | One fledgling (near full-tail) + 2 hosts |
| SUK_1123_6 | *Clamator levaillantii* | *Turdoides reinwardtii* | Sukuta (Gambia) | 01/11/2023 | Myrthe Oskampf* | Galaxy A51 phone | audio M4A | One fledgling (near full-tail) + 2 hosts |
| SUK_1123_3 | *Clamator levaillantii* | *Turdoides reinwardtii* | Sukuta (Gambia) | 03/11/2023 | Myrthe Oskampf | Galaxy A51 phone | video MP4 | One fledgling (near full-tail) + 2 hosts, sometimes showing aggression |
| [XC418749](https://xeno-canto.org/418749) | *Clamator levaillantii* | *Turdus libonyana* | Nylstroom, Bosveld, Limpopo (South Africa) | 29/01/2012 | Tony Archer | Not specified | audio MP3 | One fledgling begging from a Kurrichane Thrush (Archer 2012) |
| OLO_0723_1 | *Turdoides jardineii* | NA | Oloitokitok (Kajiado, Kenya) | 27/07/2023 | Isaac Kilusu | Phone | video MP4 | Fledgling calls (barely visible) |
| XC819613 | *Turdoides jardineii* | NA | Oloitokitok (Kajiado, Kenya) | 27/07/2023 | Isaac Kilusu | Phone | audio MP3 | Fledgling calls (barely visible) |
| XC819614 | *Turdoides jardineii* | NA | Oloitokitok (Kajiado, Kenya) | 27/07/2023 | Isaac Kilusu | Phone | audio MP3 | Fledgling calls (barely visible) |
| POL_0722_1 | *Turdoides jardineii* | NA | Polokwane (Limpopo, South Africa) | 16/07/2022 | Derek Engelbrecht | Marantz PMD 661 MK III, Sennheiser Directional microphone | audio WAV | Fledgling begging calls |
| BRU_0316_1 | *Turdoides plebejus* | NA | Brusubi (Gambia) | 22/03/2016 | Clive R. Barlow* | Marantz PMD 660, SennheiserK6 microphone, Rode 21/22 windshield | audio WAV | One fledgling + 4 adults |
| BRU_0316_2 | *Turdoides plebejus* | NA | Brusubi (Gambia) | 22/03/2016 | Clive R. Barlow* | Marantz PMD 660, SennheiserK6 microphone, Rode 21/22 windshield | audio WAV | One fledgling + 4 adults |
| BRU_0316_3 | *Turdoides plebejus* | NA | Brusubi (Gambia) | 22/03/2016 | Clive R. Barlow* | Marantz PMD 660, SennheiserK6 microphone, Rode 21/22 windshield | audio WAV | One fledgling + 4 adults |
| BRU_1021_1 | *Turdoides plebejus* | NA | Brusubi (Gambia) | 25/10/2021 | Clive R. Barlow* | Marantz PMD 660, SennheiserK6 microphone, Rode 21/22 windshield | audio WAV | One fledgling (full grown) + several adults |
| BRU_0622b_1 | *Turdoides plebejus* | NA | Brusubi (Gambia) | 28/06/2022 | Clive R. Barlow* | Marantz PMD 660, SennheiserK6 microphone, Rode 21/22 windshield | audio WAV | One fledgling + 2 adults |
| BRU_0722_1 | *Turdoides plebejus* | NA | Brusubi (Gambia) | 05/07/2022 | Clive R. Barlow* | Marantz PMD 660, SennheiserK6 microphone, Rode 21/22 windshield | audio WAV | One fledgling + 2-3 adults |
| BRU_0722_2 | *Turdoides plebejus* | NA | Brusubi (Gambia) | 06/07/2022 | Clive R. Barlow* | Marantz PMD 660, SennheiserK6 microphone, Rode 21/22 windshield | audio WAV | One fledgling + 2-3 adults |
| BRU_0623_1 | *Turdoides plebejus* | NA | Brusubi (Gambia) | 09/06/2023 | Clive R. Barlow | FujiFilm camera 24-720 mm Equity 135 30x | video MOV | One fledgling + 4 adults |
| BRU_0623_2 | *Turdoides plebejus* | NA | Brusubi (Gambia) | 14/06/2023 | Clive R. Barlow | FujiFilm camera 24-720 mm Equity 135 30x | video MOV | One fledgling + 4 adults |
| BRU_0322_1 | *Turdoides reinwardtii* | NA | Brusubi (Gambia) | 19/03/2022 | Clive R. Barlow* | Marantz PMD 660, SennheiserK6 microphone, Rode 21/22 windshield | audio WAV | One fledgling (fully mobile) + 2 adults |
| BRU_0322_2 | *Turdoides reinwardtii* | NA | Brusubi (Gambia) | 19/03/2022 | Clive R. Barlow* | Marantz PMD 660, SennheiserK6 microphone, Rode 21/22 windshield | audio WAV | One fledgling (fully mobile) + 2 adults |
| BRU_0322_3 | *Turdoides reinwardtii* | NA | Brusubi (Gambia) | 20/03/2022 | Clive R. Barlow* | Marantz PMD 660, SennheiserK6 microphone, Rode 21/22 windshield | audio WAV | One fledgling (fully mobile) + 2 adults |
| BRU_0322_4 | *Turdoides reinwardtii* | NA | Brusubi (Gambia) | 20/03/2022 | Clive R. Barlow* | Marantz PMD 660, SennheiserK6 microphone, Rode 21/22 windshield | audio WAV | One fledgling (fully mobile) + 2 adults |
| BRU_0322_5 | *Turdoides reinwardtii* | NA | Brusubi (Gambia) | 21/03/2022 | Clive R. Barlow* | Marantz PMD 660, SennheiserK6 microphone, Rode 21/22 windshield | audio WAV | One fledgling (fully mobile) + 2 adults |
| BRU_0322_6 | *Turdoides reinwardtii* | NA | Brusubi (Gambia) | 21/03/2022 | Clive R. Barlow* | Marantz PMD 660, SennheiserK6 microphone, Rode 21/22 windshield | audio WAV | One fledgling (fully mobile) + 2 adults |
| BRU_0622_1 | *Turdoides reinwardtii* | NA | Brusubi (Gambia) | 08/06/2022 | Clive R. Barlow* | Marantz PMD 660, SennheiserK6 microphone, Rode 21/22 windshield | audio WAV | One fledgling (full-grown) + 2 adults |
| BRU_0423_1 | *Turdoides reinwardtii* | NA | Brusubi (Gambia) | 02/04/2023 | Clive R. Barlow | FujiFilm camera 24-720 mm Equity 135 30x | video MOV | One fledgling + 2 adults |
| BRU_0524_1 | *Turdoides reinwardtii* | NA | Brusubi (Gambia) | 08/05/2024 | Clive R. Barlow | FujiFilm camera 24-720 mm Equity 135 30x | video MOV | One fledgling + 2 adults |
| BRU_0524_2 | *Turdoides reinwardtii* | NA | Brusubi (Gambia) | 08/05/2024 | Clive R. Barlow | FujiFilm camera 24-720 mm Equity 135 30x | video MOV | One fledgling + 2 adults |
| BRU_0524_3 | *Turdoides reinwardtii* | NA | Brusubi (Gambia) | 08/05/2024 | Clive R. Barlow | FujiFilm camera 24-720 mm Equity 135 30x | video MOV | One fledgling + 2 adults |
| BRU_0524_4 | *Turdoides reinwardtii* | *NA* | Brusubi (Gambia) | 08/05/2024 | Clive R. Barlow | FujiFilm camera 24-720 mm Equity 135 30x | video MOV | One fledgling + 2 adults |
| BRU_0822_1 | *Turdus pelios* | *NA* | Brusubi (Gambia) | 10/08/2022 | Clive R. Barlow* | Marantz PMD 660, SennheiserK6 microphone, Rode 21/22 windshield | audio WAV | One fledgling begging |
| BRU_0822_2 | *Turdus pelios* | *NA* | Brusubi (Gambia) | 10/08/2022 | Clive R. Barlow* | Marantz PMD 660, SennheiserK6 microphone, Rode 21/22 windshield | audio WAV | One fledgling begging |

**Table S2.** Predicted similarity among host begging calls based on linear discriminant analysis. Entries show, for each host species and call type (rows), the number of calls classified into each host call-type category (columns).

|  | Predicted host call type | | | | | | | | |  |  |  |
| --- | --- | --- | --- | --- | --- | --- | --- | --- | --- | --- | --- | --- |
|  | **Arrow-marked Babbler** |  | **Blackcap Babbler** | |  | **Brown Babbler** | |  | **African Thrush** |  | **N** | |
|  | **Non-transfer** |  | **Non-transfer** | **Food-transfer** |  | **Non-transfer** | **Food-transfer** |  | **Non-transfer** |  | **Calls** | **Birds** |
| Arrow-marked Babbler | |  |  |  |  |  |  |  |  |  |  |  |
| Non-transfer | 62 |  | 0 | 0 |  | 6 | 1 |  | 0 |  | 69 | 2 |
| Blackcap Babbler | |  |  |  |  |  |  |  |  |  |  |  |
| Non-transfer | 0 |  | 17 | 0 |  | 0 | 0 |  | 0 |  | 17 | 2 |
| Food-transfer | 0 |  | 0 | 5 |  | 7 | 2 |  | 0 |  | 14 | 3 |
| Brown Babbler | |  |  |  |  |  |  |  |  |  |  |  |
| Non-transfer | 17 |  | 0 | 0 |  | 183 | 6 |  | 0 |  | 206 | 3 |
| Food-transfer | 0 |  | 0 | 0 |  | 13 | 19 |  | 0 |  | 32 | 4 |
| African Thrush | |  |  |  |  |  |  |  |  |  |  |  |
| Non-transfer | 0 |  | 0 | 2 |  | 0 | 0 |  | 22 |  | 24 | 1 |

**Table S3.** Predicted similarity among cuckoo begging calls based on linear discriminant analysis. Entries show, for each host species and call type (rows), the number of calls classified into each cuckoo call-type category (columns).

|  | Predicted cuckoo call type | | | | | | | | | | |  |  |  |
| --- | --- | --- | --- | --- | --- | --- | --- | --- | --- | --- | --- | --- | --- | --- |
|  | **Arrow-marked Babbler** | |  | **Blackcap Babbler** | | |  | **Brown Babbler** | |  | **Kurrichane Thrush** |  | **N** | |
|  | **Brief non-transfer** | **Food-transfer** |  | **Brief non-transfer** | **Food-transfer** | **Meow non-transfer** |  | **Brief non-transfer** | **Food-transfer** |  | **Brief non-transfer** |  | **Calls** | **Birds** |
| Arrow-marked Babbler-cuckoo | |  |  |  |  |  |  |  |  |  |  |  |  |  |
| Non-transfer | 335 | 1 |  | 73 | 0 | 1 |  | 14 | 0 |  | 9 |  | 433 | 1 |
| Food-transfer | 7 | 0 |  | 2 | 0 | 0 |  | 0 | 0 |  | 0 |  | 9 | 1 |
| Blackcap Babbler-cuckoos | |  |  |  |  |  |  |  |  |  |  |  |  |  |
| Non-transfer | 62 | 4 |  | 66 | 1 | 1 |  | 71 | 0 |  | 0 |  | 205 | 2 |
| Food-transfer | 1 | 0 |  | 0 | 1 | 0 |  | 10 | 0 |  | 0 |  | 12 | 1 |
| Meow | 8 | 0 |  | 3 | 0 | 52 |  | 5 | 0 |  | 1 |  | 69 | 1 |
| Brown Babbler-cuckoos | | |  | |  |  |  |  |  |  |  |  |  |  |
| Non-transfer | 96 | 0 |  | 46 | 3 | 3 |  | 1266 | 2 |  | 7 |  | 1423 | 3 |
| Food-transfer | 1 | 0 |  | 0 | 0 | 0 |  | 26 | 4 |  | 0 |  | 31 | 1 |
| Kurrichane Thrush-cuckoo | | |  | |  |  |  |  |  |  |  |  |  |  |
| Non-transfer | 2 | 0 |  | 0 | 0 | 0 |  | 2 | 0 |  | 3 |  | 7 | 1 |

**Table S4.** Predicted similarity of begging calls based on the logistic regression functions for each cuckoo–host pair. Entries show, for each call type and host association (rows), the number of cuckoo calls classified into each host call-type category (columns). The F1 score and Matthews correlation coefficient (MCC) provide call-type–independent measures of overall similarity between each cuckoo and its associated host relative to other hosts

|  | Associated host | | | | | | | | | |  |  |  |
| --- | --- | --- | --- | --- | --- | --- | --- | --- | --- | --- | --- | --- | --- |
|  | **Arrow-marked Babbler** | |  | **Blackcap Babbler** | |  | **Brown Babbler** | |  | **African Thrush** |  |  |  |
|  | **Non-transfer** | |  | **Non-transfer** | **Food-transfer** |  | **Non-transfer** | **Food-transfer** |  | **Non-transfer** |  | **F1^1^** | **MCC^2^** |
| Arrow-marked Babbler cuckoo | | | | | |  |  |  |  |  |  | 0.09 | 0.14 |
| Non-transfer | 22 | |  | 26 | 6 |  | 364 | 15 |  | 0 |  |  |  |
| Food-transfer | 0 | |  | 0 | 0 |  | 8 | 1 |  | 0 |  |  |  |
| Blackcap Babbler cuckoos | |  | | |  |  |  |  |  |  |  | 0.11 | -0.09 |
| Non-transfer | 5 | |  | 1 | 15 |  | 180 | 4 |  | 1 |  |  |  |
| Food-transfer | 0 | |  | 0 | 5 |  | 7 | 0 |  | 0 |  |  |  |
| Meow | 3 | |  | 23 | 5 |  | 19 | 0 |  | 19 |  |  |  |
| Brown Babbler cuckoos | |  | | |  |  |  |  |  |  |  | 0.62 | -0.19 |
| Non-transfer | 4 | |  | 14 | 478 |  | 912 | 12 |  | 3 |  |  |  |
| Food-transfer | 0 | |  | 0 | 26 |  | 2 | 0 |  | 3 |  |  |  |
| Kurrichane Thrush cuckoo | |  | | |  |  |  |  |  |  |  | 0.00 | -0.01 |
| Non-transfer | 0 | |  | 0 | 1 |  | 4 | 2 |  | 0 |  |  |  |

^1^ 0 (complete classification failure) < F1 < 1 (perfect classification)

^2^ –1 (poor) < MCC < 1 (perfect), with 0 indicating random performance. MCC >0.3 is often considered moderate and >0.5 strong

**Table S5.** Predicted similarity of begging calls based on the logistic regression functions for each cuckoo–host pair. Entries show, for each call type and host association (rows), the number of cuckoo calls classified as their associated host or as cuckoos associated with other hosts (columns). The F1 score and Matthews correlation coefficient (MCC) provide call-type–independent measures of similarity between each cuckoo and its associated host relative to other cuckoos.

|  |  | | Cuckoos | | | | | | | | | | | |  |  |  |
| --- | --- | --- | --- | --- | --- | --- | --- | --- | --- | --- | --- | --- | --- | --- | --- | --- | --- |
|  | **Associated host** | |  | **Arrow-marked Babbler** | |  | **Blackcap Babbler** | | |  | **Brown Babbler** | |  | **Kurrichane** **Thrush** |  |  |  |
|  | **Brief non-transfer** | **Food-transfer** |  | **Brief non-transfer** | **Food-transfer** |  | **Brief non-transfer** | **Food-transfer** | **Meow non-transfer** |  | **Brief non-transfer** | **Food-transfer** |  | **Brief non-transfer** |  | **F1** | **MCC** |
| Arrow-marked Babbler-cuckoo | | | | | |  |  |  |  |  |  |  |  |  |  | 0.21 | 0.07 |
| Brief non-transfer | 77 | -- |  | -- | -- |  | 149 | 0 | 77 |  | 130 | 0 |  | 0 |  |  |  |
| Food-transfer | 2 | -- |  | -- | -- |  | 4 | 0 | 0 |  | 3 | 0 |  | 0 |  |  |  |
| Blackcap Babbler-cuckoos | | | | |  |  |  |  |  |  |  |  |  |  |  | 0.02 | -0.20 |
| Brief non-transfer | 0 | 1 |  | 78 | 3 |  | -- | -- | -- |  | 123 | 1 |  | 0 |  |  |  |
| Food-transfer | 0 | 0 |  | 1 | 0 |  | -- | -- | -- |  | 11 | 0 |  | 0 |  |  |  |
| Meow non-transfer | 7 | 2 |  | 22 | 0 |  | -- | -- | -- |  | 11 | 0 |  | 27 |  |  |  |
| Brown Babbler-cuckoos | | | | |  |  |  |  |  |  |  |  |  |  |  | 0.73 | 0.30 |
| Brief non-transfer | 934 | 35 |  | 104 | 0 |  | 309 | 18 | 14 |  | -- | -- |  | 9 |  |  |  |
| Food-transfer | 28 | 2 |  | 0 | 0 |  | 1 | 0 | 0 |  | -- | -- |  | 0 |  |  |  |
| Kurrichane Thrush-cuckoo | | | | |  |  |  |  |  |  |  |  |  |  |  |  |  |
| Brief non-transfer | 0 | -- |  | 1 | 0 |  | 0 | 0 | 1 |  | 5 | 0 |  | -- |  | 0.00 | -0.01 |

^1^ 0 (complete classification failure) < F1 < 1 (perfect classification)

^2^ –1 (poor) < MCC < 1 (perfect), with 0 indicating random performance. MCC >0.3 is often considered moderate and >0.5 strong
